# Cyclophilin D Contributes to Anesthesia Neurotoxicity in the Developing Brain

**DOI:** 10.3389/fcell.2019.00396

**Published:** 2020-02-11

**Authors:** Yiying Zhang, Pan Lu, Feng Liang, Ning Liufu, Yuanlin Dong, Jialin Charles Zheng, Zhongcong Xie

**Affiliations:** ^1^Center for Neuroimmunology and Regenerative Therapy, Shanghai Tenth People’s Hospital, Anesthesia and Brain Research Institute, Tongji University School of Medicine, Shanghai, China; ^2^Department of Anesthesia, Critical Care and Pain Medicine, Harvard Medical School, Massachusetts General Hospital, Charlestown, MA, United States; ^3^Department of Pharmacology and Experimental Neurosciences, Nebraska Medical Center, University of Nebraska Medical Center, Omaha, NE, United States

**Keywords:** anesthesia, sevoflurane, mitochondrial function, cyclophilin D, neurogenesis, cognition, young mice

## Abstract

Anesthetic sevoflurane induces mitochondrial dysfunction, impairment of neurogenesis, and cognitive impairment in young mice, but the underlying mechanism remains to be determined. Cyclophilin D (CypD) is a modulatory factor for the mitochondrial permeability transition pore (mPTP). We, therefore, set out to evaluate the role of CypD in these sevoflurane-induced changes *in vitro* and in young mice. Wild-type (WT) and CypD knockout (KO) young (postnatal day 6, 7, and 8) mice received 3% sevoflurane 2 h daily and the neural progenitor cells (NPCs) harvested from the WT or CypD KO mice received 4.1% sevoflurane. We used immunohistochemistry and immunocytochemistry imaging, flow cytometry, Western blot, RT-PCR, co-immunoprecipitation, and Morris Water Maze to assess the interaction of sevoflurane and CypD on mitochondria function, neurogenesis, and cognition *in vitro* and in WT or CypD KO mice. We demonstrated that the sevoflurane anesthesia induced accumulation of CypD, mitochondrial dysfunction, impairment of neurogenesis, and cognitive impairment in WT mice or NPCs harvested from WT mice, but not in CypD KO mice or NPCs harvested from CypD KO mice. Furthermore, the sevoflurane anesthesia reduced the binding of CypD with Adenine nucleotide translocator, the other component of mPTP. These data suggest that the sevoflurane anesthesia might induce a CypD-dependent mitochondria dysfunction, impairment of neurogenesis, and cognitive impairment in young mice and NPCs.

## Introduction

Anesthesia has been reported to impair mitochondrial functions in the brain of young rodents (reviewed in Vutskits and Xie, 2016). Sanchez et al. (2011) demonstrated that midazolam, nitrous oxide, and isoflurane enlarged mitochondria size, impaired structural integrity of mitochondria, and affected complex IV activity. Consistently, Boscolo et al. (2013) found that the same anesthetics increased brain levels of ROS and impaired balance between mitochondrial fission and fusion, leading to excessive fission, and impaired mitochondrial morphogenesis (Boscolo et al., 2013). Lunardi et al. (2010) also showed that these anesthetics induced mitochondria degeneration. In addition, Sun et al. (2016) reported that NADPH oxidase inhibitor apocynin attenuated the sevoflurane-induced cognitive impairment and the increases in brain levels of superoxide and NADPH oxidase subunit p22phox. ROS scavenger inhibited the anesthesia-induced mitochondrial dysfunction and cognitive impairment in rats (Boscolo et al., 2012). Moreover, our previous studies showed that anesthetic isoflurane caused the opening of mPTP (Zhang et al., 2010, 2012b).

Sevoflurane, the most common anesthetic in children, reduced activity of NADH:ubiquinone oxidoreductase in mitochondria (Hanley et al., 2005), decreased MMP (Xu et al., 2017), induced mitochondria hyperactivity (Chung et al., 2017), reduced mitochondria function (Amrock et al., 2015), and decreased ATP levels (Xu et al., 2017) in young rodents. However, the underlying mechanism and the consequences by which anesthesia induces mitochondrial dysfunction remains largely to be determined.

Anesthesia has been reported to impair neurogenesis in developing brain (Stratmann et al., 2009; Zhu et al., 2010; Shen et al., 2013; Zhang et al., 2013; Yi et al., 2016; Fang et al., 2017). Sevoflurane inhibited neurogenesis of hippocampus neural stem cells *in vitro* and in rodent models (Engelhard et al., 2007; Zhang et al., 2015; Yi et al., 2016; Fang et al., 2017), and in human embryonic stem cells (Park et al., 2017). However, the underlying mechanism remains mostly unknown.

Cyclophilin D, coding by Ppif gene, is a protein located in the matrix of mitochondria, a component of the mPTP and a crucial modulatory factor for mPTP function (Baines et al., 2005; Basso et al., 2005), which binds to ANT, another component of mPTP (Duarte et al., 2013). The expression of CypD was elevated in brain tissues of Alzheimer’s disease (AD) transgenic mice (Du et al., 2008, 2011, 2014), which promotes AD neuropathogenesis (Wang et al., 2009; Zhu et al., 2013; Adiele and Adiele, 2016). Knockout (KO) of CypD stabilized mitochondrial function (Du et al., 2008, 2011, 2014; Gainutdinov et al., 2015; Gordan et al., 2016; Sun and Jacobs, 2016), decreased threshold of mPTP formation, and improved cognitive function in old AD transgenic mice (Du et al., 2008, 2011, 2014). However, the role of CypD in the developmental anesthesia neurotoxicity remains largely to be determined.

We, therefore, set out to determine whether anesthetic sevoflurane could induce a CypD-dependent mitochondrial dysfunction, impairment of neurogenesis, and cognitive impairment in young mice and in NPCs by testing a hypothesis that KO of CypD mitigated the sevoflurane-induced mitochondrial dysfunction, impairment of neurogenesis, and cognitive impairment in NPC and in young mice.

Notably, many investigators have performed the research of anesthesia and mitochondria. The objective of the current study, however, was to specifically determine the role of CypD in the anesthesia-induced mitochondrial dysfunction. The experiments to investigate the mitochondrial function (e.g., mPTP, ROS, and MMP) in the current studies were only used to demonstrate the role of CypD in the anesthesia-induced mitochondrial dysfunction.

## Materials and Methods

### Mice Anesthesia

The protocol of the animal study was approved by the Massachusetts General Hospital Standing Committee on Animals (Boston, MA, United States) on the Use of Animals in Research and Teaching. We performed animal studies according to the regulation of the National Institutes of Health (Bethesda, MD, United States). Both female and male (both sex) C57BL/6J WT and B6;129-Ppiftm1Maf/J, Ppif−/− CypD KO young mice (Jackson Laboratory, Bar Harbor, ME, United States) were used in the experiments. See Supplementary Material for details.

### 5′-Bromo-2′-Deoxyuridine (BrdU) Injection for the Assessment of Neurogenesis

Each of the young mice received intraperitoneal (IP) injections of BrdU at a dose of 50 mg/kg body weight to label dividing cells 30 min before each of the sevoflurane anesthesia (Bartley et al., 2005; Wei et al., 2018). See Supplementary Material for details.

### Harvest of Brain Tissues

The mice were decapitated on postnatal day 8 (P8) or P36, and the brain hippocampus tissues were harvested. See Supplementary Material for details.

### Mitochondria Isolation

We used Mitochondria Isolation Kits for tissue (Thermo Fisher Scientific, Waltham, MA, United States) to isolate the mitochondria using the protocol provided by the company. See Supplementary Material for details.

### Western Blots Analysis

Antibodies used in the experiments were anti-CypD (1:1000, ab110324, Abcam, Cambridge, MA, United States); anti-VDAC1 (1:1000, ab154856, Abcam); anti-ANT (1:1000, ab180715, Abcam); and anti-β-Actin (1:10,000, Sigma, St. Louis, MO, United States). See Supplementary Material for details.

### Reactive Oxygen Species (ROS) Measurement

We used OxiSelect Intracellular ROS Assay Kit (Cell Biolvitro, STA-342) to detect ROS in NPCs, and employed OxiSelect *in vitro* ROS/RNS Assay Kit (Cell Biolvitro, STA-347) to measure ROS in the brain tissues of young mice as performed in our previous studies (Zhang et al., 2012b). See Supplementary Material for details.

### Measurement of Mitochondrial Membrane Potential (MMP)

We used JC-1 MMP detection kit (Biotium Inc., Fremont, CA, United States) to determine MMP levels (Zhang et al., 2012b). See Supplementary Material for details.

### ATP Measurement

We employed the ATP Assay Kit (Colorimetric/Fluorometric) (Abcam) to detect ATP levels (Zhang et al., 2012b). See Supplementary Material for details.

### Sectioning of Fresh Unfixed Tissue Using a Compresstome

We used half of the brain for tissue immunofluorescence staining and half of the brain for Western blot analysis. This post-harvest fixation technique was based on the methods described by Abdelaal et al. (2015). See Supplementary Material for details.

### Immunohistochemistry

Immunohistochemistry was used to assess the effects of sevoflurane on number of BrdU- and BrdU/Nestin-double positive cells in mouse brain tissue (Drobish et al., 2016). See Supplementary Material for details.

### NPCs Culture

We harvested NPCs using the methods described in our previous studies (Zhang et al., 2013, 2014). See Supplementary Material for details.

### NPCs and H4 Cells Culture and Treatment

We performed NPCs and H4 cells studies using the methods described in our previous studies (Zhang et al., 2013, 2014). See Supplementary Material for details.

### Labeling NPCs Using EdU and Quantification

Click-iT^TM^ EdU Flow Cytometry Assay Kit (Invitrogen, St. Louis, MO, United States) was used in the experiments to detect EdU-positive cells (Zhang et al., 2013, 2014). See Supplementary Material for details.

### Flow Cytometric Analysis of mPTP Opening

Opening of mPTP was determined by flow cytometry, using the MitoProbe Transition Pore Assay Kit (Invitrogen) (Zhang et al., 2012a, b). See Supplementary Material for details.

### Reverse Transcriptase Polymerase Chain Reaction (RT-PCR)

Real-time One-Step RT-PCR was carried out using the QuantiTect SYBR Green real-time polymerase chain reaction kit (Qiagen, Valencia, CA, United States). See Supplementary Material for details.

### Immunocytochemistry Staining

For detection of colocalization of CypD and ANT, H4 cells were analyzed in mounting medium under a 100 × objective lens fluorescence microscope, using the methods described in our previous studies (Zhang et al., 2013). See Supplementary Material for details.

### Co-immunoprecipitation of CypD and ANT

The co-immunoprecipitation studies were performed using the methods described before (Duarte et al., 2013). See Supplementary Material for details.

### Morris Water Maze (MWM) Studies

Morris Water Maze was performed as described before (Shen et al., 2013; Tao et al., 2014; Zhang et al., 2015; Lu et al., 2017) with modifications. See Supplementary Material for details.

### Statistical Analysis

We performed a power analysis based on the data obtained from the previous studies (Tao et al., 2014). The data obtained from our biochemistry studies and the escape latency of MWM were presented as mean ± SD or mean ± standard error of the mean (SEM). One-way or two-way ANOVA and Student’s *t* test, with *post-hoc* Bonferroni correction, and Mann–Whitney test were used in the studies with *P* values less than 0.05 considered statistically significant. See Supplementary Material for details. The numbers of the platform crossing time of MWM were not normally distributed. Hence, the data were presented as the median with interquartile ranges. The number of samples was 10 per group in the behavioral studies; 6 samples per group for the Western blot, ROS, ATP, and fluorescence staining studies; and 3 samples per group for the flow cytometry studies, MMP studies, and RT-PCR studies. Interaction between time and group factors was determined by using a two-way ANOVA with repeated measurements to analyze the difference in learning curves (based on escape latency) between mice in the control group and mice treated with sevoflurane anesthesia in the MWM. A Student’s *t* test with *post hoc* Bonferroni correction were used to compare the difference in escape latency between the control condition and the anesthesia group during each day of the MWM. The Mann–Whitney test was used to determine the difference between the control condition and sevoflurane anesthesia in terms of the platform crossing times. There were no missing data for the variables of the MWM (escape latency and platform crossing times) during the data analysis. A one-way ANOVA with Bonferroni comparison was used to determine the differences among multiple groups. Finally, a Student’s *t* test with Bonferroni *post hoc* correction was used to compare the difference between the two groups. *P* values less than 0.05 were considered statistically significant, and the significance testing was two-tailed. Statistical analysis was conducted using the GraphPad Prism software (version 8.0).

## Results

### Sevoflurane Increased CypD Levels in Mice Brain Tissues

**FIGURE 1:**
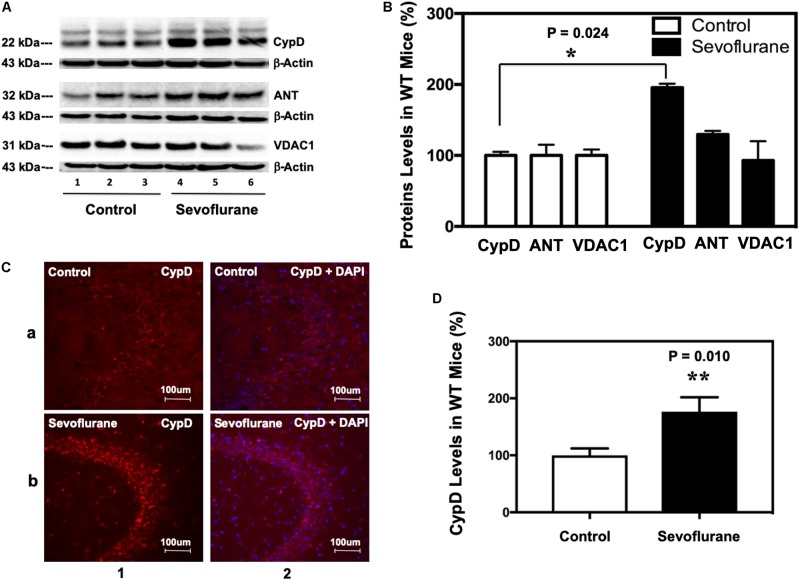
Sevoflurane increases the levels of CypD, but not ANT or VDAC, in hippocampus tissues of WT young mice. **(A)** Western blot shows that sevoflurane anesthesia (lanes 4–6) increases the levels of CypD, but not ANT or VDAC, other two components of mPTP, as compared to the control condition (lanes 1–3) in the hippocampus tissues of WT mice. There is no significant difference in the amounts of β-actin in the hippocampus tissues between the mice in the control condition group and the mice in the sevoflurane anesthesia group. **(B)** Quantification of the Western blot shows that the sevoflurane anesthesia (black bar) increases CypD levels as compared to the control condition (white bar). (**P = 0.024, Student’s *t* test with *post hoc* Bonferroni adjustment, *N* = 6). **(C)** Sevoflurane anesthesia (row b) increases the levels of CypD compared to the control condition (row a) in the hippocampus tissues of WT mice. Column 1 is the CypD (red) staining, and column 2 is CypD merged with the DAPI (blue) nuclear staining. **(D)** Quantification of the image shows that sevoflurane anesthesia (black bar) increases CypD levels as compared to the control condition (white bar). (**P = 0.010, Student’s *t* test, *N* = 6).

Immunoblotting of CypD revealed that sevoflurane anesthesia increased the levels of CypD, but not ANT or VDAC, the other two components of mPTP, as compared to the control condition in hippocampus of P8 (Figure 1A), but not P36, mice (data not shown). Quantification of the Western blot, based on the ratio of CypD level to β-actin level, demonstrated that the sevoflurane anesthesia increased CypD levels in mice hippocampus: 187 ± 15% versus 100 ± 13%, ^∗^*P* = 0.024 (Figure 1B). Immunofluorescence staining also showed that sevoflurane anesthesia increased CypD levels (Figures 1C,D).

### Sevoflurane Induced a CypD-Dependent Mitochondrial Dysfunction in Young Mice

**FIGURE 2:**
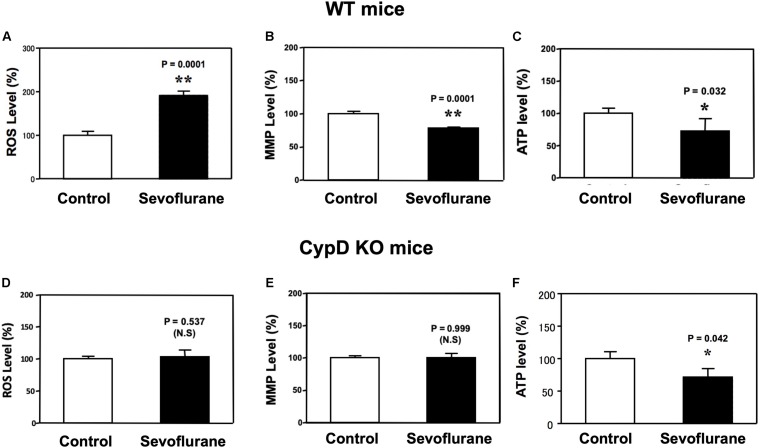
Sevoflurane induces a CypD-dependent mitochondrial dysfunction in hippocampus tissues of young mice. **(A)** Sevoflurane anesthesia (black bar) increases ROS levels as compared to the control condition (white bar) in hippocampus tissues of WT mice (**P = 0.001, Student’s *t* test, *N* = 6). **(B)** Tetraethylbenzimidazolylcarbocyanine iodide fluorescence analysis shows that sevoflurane anesthesia (black bar) reduces levels of MMP as compared to the control condition in hippocampus tissues of WT mice (**P = 0.001, Student’s *t* test, *N* = 3). **(C)** Sevoflurane anesthesia (black bar) decreases ATP levels as compared to the control condition (white bar) in the hippocampus tissues of WT mice. (*P = 0.032, Student’s *t* test, *N* = 6). **(D)** Sevoflurane anesthesia (black bar) does not significantly change the ROS levels as compared to the control condition (white bar) in the hippocampus tissues of CypD KO mice (*P* = 0.537, Student’s *t* test, *N* = 6). **(E)** Sevoflurane anesthesia (black bar) does not significantly change the levels of MMP as compared to the control condition in hippocampus tissues of CypD KO mice (*P* = 0.999, Student’s *t* test, *N* = 3). **(F)** Sevoflurane anesthesia (black bar) decreases ATP levels as compared to the control condition (white bar) in CypD KO mice hippocampus tissues. (*P = 0.042, Student’s *t* test, *N* = 6). N.S., not significant.

Sevoflurane anesthesia increased ROS levels and reduced MMP levels in hippocampus of WT (Figures 2A,B), but not CypD KO, mice (Figures 2D,E) as compared to the control condition. Interestingly, the sevoflurane anesthesia reduced hippocampus ATP levels in both WT and CypD KO mice (Figures 2C,F). There was no significant difference in the baseline levels of ROS between the brain tissues of WT mice and the brain tissues of the CypD KO mice: 441.3 (nM) versus 460.5 (nM), *P* = 0.680 (Supplementary Figure 1A).

### Sevoflurane Induced a CypD-Dependent Impairment of Neurogenesis in Young Mice

**FIGURE 3:**
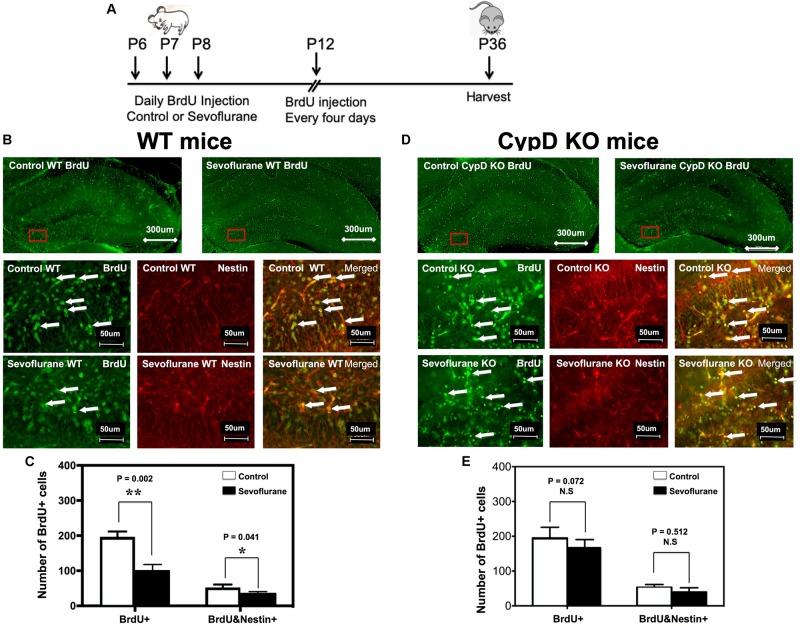
Sevoflurane anesthesia induces a CypD-dependent impairment of neurogenesis in hippocampus tissues of young mice. **(A)** The diagram showing the schedule of BrdU injection and sevoflurane anesthesia. BrdU injection was administered 30 min before the sevoflurane anesthesia on P6, P7, and P8. We then repeated the BrdU administration every 4 days. The mice hippocampus tissues were harvested at P8 or P36 for immunohistochemistry staining. **(B)** Immunohistochemistry staining for BrdU and Nestin in hippocampus tissues of WT mice. The WT mice in the sevoflurane anesthesia group (top right panel and bottom panels) showed fewer numbers of BrdU-positive cells and BrdU/Nestin double-positive cells in the DG compared to the WT mice in the control condition group (top left panel and middle panels). **(C)** Quantification of BrdU-positive cells and BrdU/Nestin double-positive cells in each group. Values represent data from four independent experiments in the WT mice (for the BrdU-positive cells, **P = 0.002, Student’s *t* test, *N* = 6; for the BrdU/Nestin double-positive cells, *P = 0.041, Student’s *t* test, *N* = 6). **(D)** In the hippocampus tissues of CypD KO mice, there are no significant differences in the BrdU-positive cells or BrdU/Nestin double-positive cells in the DG between the mice in the control condition group (top left panel and middle panels) and the mice in the sevoflurane anesthesia group (top right panel and bottom panels). **(E)** Quantification of BrdU-positive cells and BrdU/Nestin double-positive cells in CypD KO mice (BrdU-positive cells, *P* = 0.072, Student’s *t* test, *N* = 6; BrdU/Nestin double-positive cells, *P* = 0.512, Student’s *t* test, *N* = 6). N.S., not significant.

The timeline of BrdU injection was demonstrated in Figure 3A. Immunofluorescence staining demonstrated the BrdU and Nestin staining in WT (Figure 3B) and CypD KO **(3D)** mice. Here, we showed that there were fewer BrdU-positive cells, both the number and the ratio, in P8 WT mice (Figures 3B,C and Supplementary Figure 2A), but not in CypD KO mice (Figures 3D,E and Supplementary Figure 2B), following the administration of the sevoflurane anesthesia as compared to the control condition. Moreover, we demonstrated that there were fewer BrdU/Nestin-double positive cells in the inner granular layer (IGL) and outer granular layer (OGL) of DG in the P8 WT mice (Figures 3B,C), but not in the P36 WT mice (data not shown) or in the CypD KO mice (Figures 3D,E) following the administration of the sevoflurane anesthesia as compared to the control condition.

### Sevoflurane Induced a CypD-Dependent Cognitive Impairment in Young Mice

**FIGURE 4:**
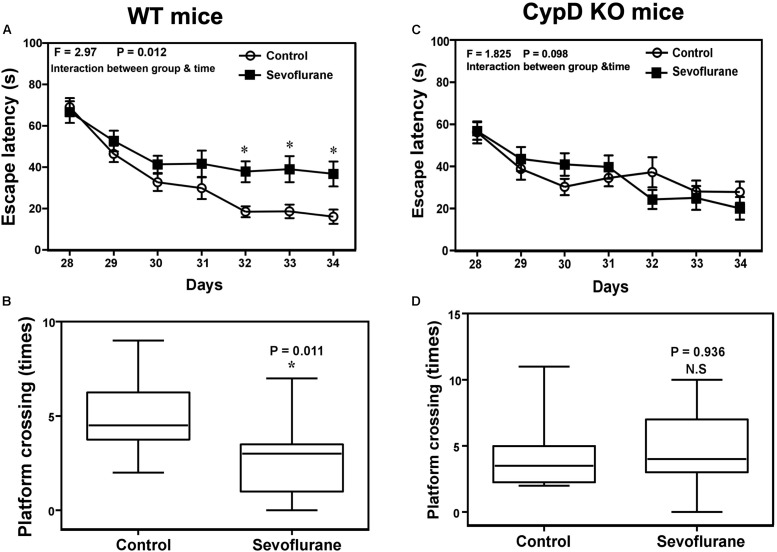
Sevoflurane induces a CypD-dependent cognitive impairment in young mice. **(A)** Two-way ANOVA with repeated measurement analysis shows that there is significant interaction between the treatment (control condition and sevoflurane anesthesia) and time (P28 to P34) on escape latency (*N* = 10 in each group, *F* = 2.970, *P = 0.012) in the WT mice. The *post hoc* (Bonferroni) test shows that the mice following the sevoflurane anesthesia have longer escape latency than the mice following the control condition at P32, P33, and P34. **(B)** The Mann–Whitney test shows that the WT mice following the sevoflurane anesthesia have less platform crossing times than the WT mice following the control condition (*N* = 10 in each group, *P* = 0.011). **(C)** Two-way ANOVA with repeated measurement analysis shows that there is no significant interaction between the treatment (control condition and sevoflurane anesthesia) and time (P28 to P34) on escape latency (*N* = 10 in each group, *F* = 1.825, *P* = 0.098) in the CypD KO mice. **(D)** The Mann–Whitney test shows that there is no significant difference in platform crossing times between the CypD KO mice following the sevoflurane anesthesia and the CypD KO mice following the control condition (*N* = 10 in each group, *P* = 0.936). N.S., not significant.

Two-way ANOVA with repeated measurement showed a statistically significant interaction of time (P28 to P34) and treatment (sevoflurane anesthesia vs. control condition) on the escape latency in MWM (Figure 4A, *F* = 2.970, ^∗^*P* = 0.012). The *post hoc* Bonferroni correction showed that the mice that received the sevoflurane anesthesia had longer escape latency than the mice following the control condition on P32, P33, and P34. Sevoflurane anesthesia decreased the platform crossing times as compared to the control condition (Figure 4B, ^∗^*P* = 0.011, Mann–Whitney test). However, the same sevoflurane anesthesia did not significantly change the escape latency or reduce the platform crossing times as compared to the control condition in CypD KO mice. There was no significant difference in swimming speed between the mice in the sevoflurane anesthesia and the control condition (data not shown).

### Sevoflurane Induced a CypD-Dependent Reduction in Proliferation of NPCs in Cell Culture

**FIGURE 5:**
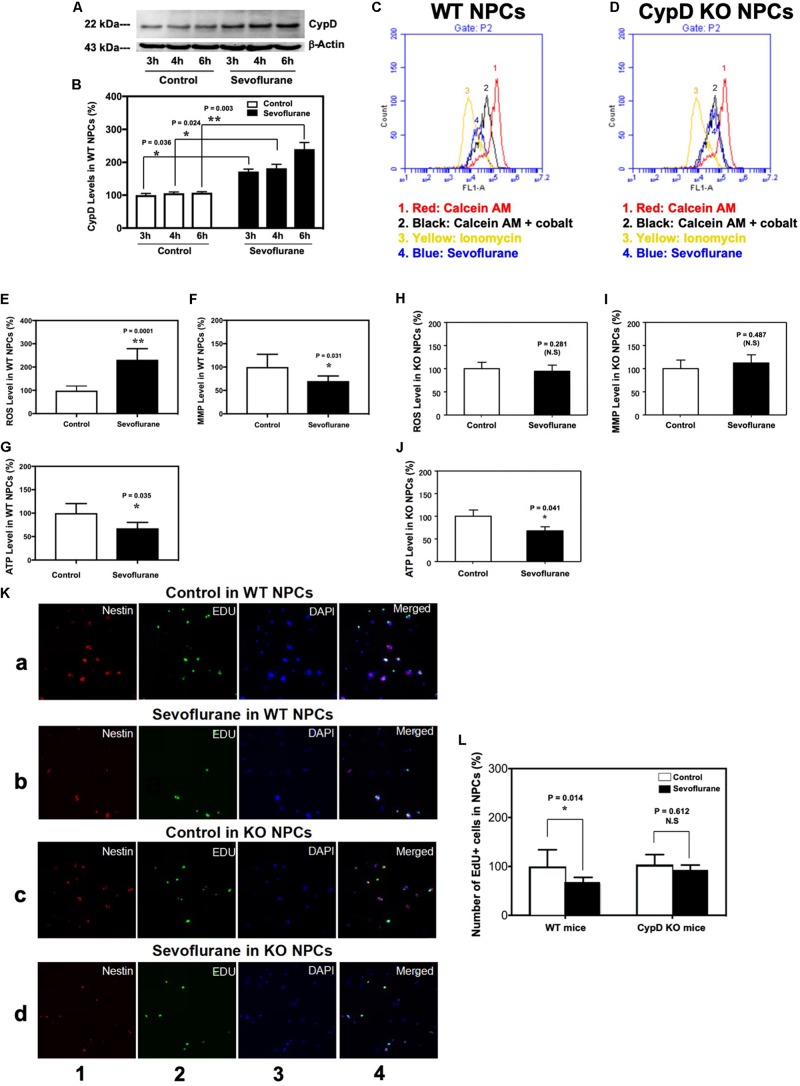
Sevoflurane induces a CypD-dependent mitochondrial dysfunction and impairment of neurogenesis in NPCs. **(A)** Western blot shows a time-dependent increase of CypD levels after sevoflurane anesthesia as compared to the control condition in the WT NPCs. There is no significant difference in the amount of β-actin in each group. **(B)** Quantification of the Western blot shows that sevoflurane anesthesia (black bar) increases CypD levels as compared to the control condition in the WT NPCs (white bar). (*P = 0.036, *P = 0.024, and **P = 0.003, Student’s *t* test with *post hoc* Bonferroni adjustment, *N* = 6). **(C)** Flow cytometric analysis shows changes in the calcein levels in mitochondria of WT NPCs, which indicates the opening of mPTP: peak 1, calcein AM-treated NPCs; peak 2, negative control (treatment of calcein AM plus cobalt); peak 3, positive control (treatment of calcein AM plus cobalt and ionomycin); peak 4, calcein AM plus cobalt and sevoflurane. The changes in the intensity of fluorescence between sevoflurane anesthesia (peak 4), positive control (peak 3), and negative control (peak 2) suggest that sevoflurane induces opening of mPTP. **(D)** Flow cytometric analysis shows changes in the calcein levels in mitochondria of CypD KO NPCs, which indicates that sevoflurane does not induce the opening of mPTP. **(E)** Sevoflurane anesthesia (black bar) increases ROS levels as compared to the control condition (white bar) in WT NPCs. (**P = 0.0001, Student’s *t* test, *N* = 6). **(F)** Sevoflurane anesthesia (black bar) reduces levels of MMP as compared to the control condition in WT NPCs (*P = 0.031, Student’s *t* test, *N* = 6). **(G)** Sevoflurane (black bar) decreases ATP levels as compared to the control condition (white bar) in WT NPCs. (*P = 0.035, Student’s *t* test, *N* = 6). **(H)** Sevoflurane anesthesia does not significantly affect the ROS levels in CypD KO NPCs. **(I)** Sevoflurane anesthesia does not significantly affect the MMP levels in CypD KO NPCs. **(J)** Sevoflurane anesthesia (black bar) decreases ATP levels as compared to the control condition (white bar) in the CypD KO NPCs. (*P = 0.041, Student’s *t* test, *N* = 6). **(K)** Immunocytochemistry image shows that the sevoflurane anesthesia (row b) decreases the number of EdU-positive and EdU/Nestin-positive cells as compared to the control condition (row a) in WT NPCs. However, sevoflurane anesthesia (row d) does not significantly change the number of EdU-positive cells and EdU/Nestin-positive cells as compared to control condition (row c) in the CypD KO NPCs. Column 1 is the image of Nestin staining (red), the marker of NPCs; column 2 is EdU staining (green), the marker of proliferated cells; column 3 is nuclei staining (blue), and column 4 is the merged image. The purple color in column 4 indicates the proliferated NPCs. **(L)** Flow cytometric analysis shows that the sevoflurane anesthesia decreases the number of EdU-positive cells as compared to the control condition (white bar) in the WT NPCs (*P = 0.014, Student’s *t* test, *N* = 3), but not in the CypD KO NPCs (*P* = 0.612, Student’s *t* test, *N* = 3). N.S., not significant.

In the *in vivo* studies described in the previous sections, the mice received 60% oxygen, which could be a confounding factor in the studies. We, therefore, performed the *in vitro* studies using 21% oxygen to further identify the role of CypD in the sevoflurane-induced mitochondrial dysfunction and impairment of neurogenesis. The immunoblotting of CypD showed that the sevoflurane anesthesia led to a time-dependent increase in CypD levels as compared to the control condition in the WT NPCs (Figures 5A,B). There was no significant difference in β-actin levels between the control condition and the sevoflurane anesthesia.

Flow cytometric analysis showed that the sevoflurane anesthesia induced opening of mPTP as compared to the control condition in WT NPCs (Figure 5C), but not in CypD KO NPCs (Figure 5D). Sevoflurane increased ROS levels (Figure 5E) and reduced levels of MMP (Figure 5F) in the WT NPCs, but not in the CypD KO NPCs (Figures 5H,I). The sevoflurane anesthesia reduced ATP levels in both WT and CypD KO NPCs (Figures 5G,J).

Finally, immunocytochemistry staining showed that there were decreases in the number of EdU-positive cells and EdU/Nestin-double positive cells following the sevoflurane anesthesia as compared to the control condition in WT NPCs, but not in the CypD KO NPCs (Figure 5K). Flow cytometry studies also demonstrated significant decreases in the proliferation of WT NPCs, but not CypD KO NPCs, following sevoflurane (Figure 5L).

### Sevoflurane Decreased the Binding of CypD With ANT

**FIGURE 6:**
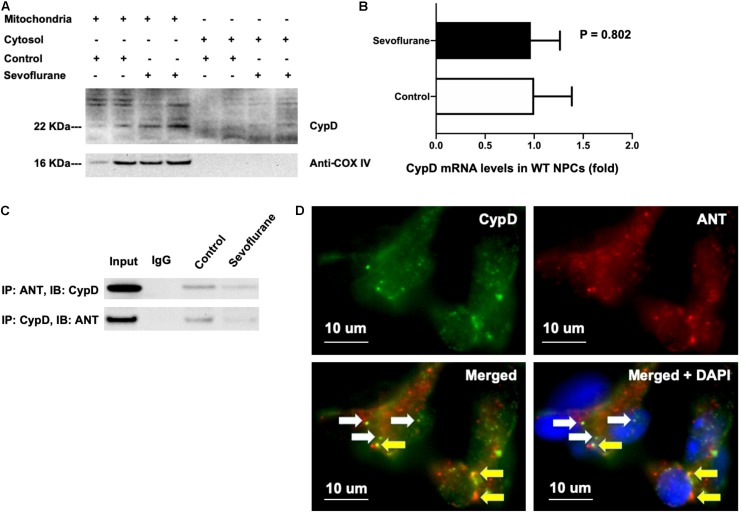
Sevoflurane anesthesia reduces binding of CypD and ANT. **(A)** CypD mainly locates in isolated mitochondria, but not cytosol, and its level can be increased in the isolated mitochondria of WT NPCs following the sevoflurane anesthesia. **(B)** RT-PCR shows that sevoflurane anesthesia does not significantly increase the mRNA levels of CypD as compared to the control condition in WT NPCs (*P* = 0.802, Student’s *t* test, *N* = 3). **(C)** Co-immunoprecipitation studies show that sevoflurane anesthesia reduces the binding of CypD with ANT as compared to the control condition in H4 human neuroglioma cells. **(D)** Immunocytochemistry images show that there are both CypD bound with ANT (yellow arrow) and CypD separated with ANT (white arrow) in the H4 human neuroglioma cells following sevoflurane anesthesia. Specifically, the green dots demonstrated CypD (**D**, top left panel), and the red dots showed ANT (**D**, top right panel). The white arrows in the bottom two panels of **D** indicated the CypD that did not bind to ANT, and the yellow arrows in the bottom panels of **D** revealed the CypD that bonded to ANT.

Cyclophilin D and adenine nucleotide translocase bind to each other in the normal physiology condition (Duarte et al., 2013). We found that most CypD existed in the isolated mitochondria but not in the isolated cytosol, and the sevoflurane anesthesia increased CypD levels as compared to the control condition in the isolated mitochondria (Figure 6A). Note that we used anti-cox IV in Figure 6A as the loading control and also as the indication that the harvested tissues were indeed the mitochondria. In the isolated cytosol, we purposely did not include any control protein because we wanted to show that the harvested tissues were not mitochondria, as evidenced by the absence of anti-cox IV, the structure protein of mitochondria. Sevoflurane did not increase mRNA levels of CypD as compared to the control condition in NPCs (Figure 6B). Here, we used H4 cells instead of NPCs because we could harvest larger amounts of the H4 cells for the co-immunoprecipitation studies. Finally, the sevoflurane anesthesia reduced the binding between CypD and ANT in H4 cells (Figure 6C). We qualitatively illustrated co-localization of CypD and ANT in the H4 cells following the sevoflurane anesthesia (Figure 6D). Taken together, these data suggest that sevoflurane might increase the levels of CypD via reducing the binding between CypD and ANT, but not via the increasing the generation of CypD, pending further confirmative investigation.

## Discussion

The objective of the current study was not to study the general vulnerability of mitochondria following the administration of anesthesia, but rather to reveal the specific role of CypD in mitochondrial function, neurogenesis, and cognition *in vivo* in young mice and *in vitro* in NPCs following sevoflurane anesthesia.

We found that the sevoflurane anesthesia specifically increased protein levels of CypD in hippocampus of young mice and NPCs (Figures 1, 5). We demonstrated that sevoflurane anesthesia induced mitochondrial dysfunction, decreased proliferation of NPCs, impaired neurogenesis, and caused cognitive impairment in WT young mice and WT NPCs, but not in CypD KO young mice or CypD KO NPCs (Figures 2–5). Finally, we performed studies to show that sevoflurane anesthesia might increase mitochondrial levels of CypD by releasing the binding of CypD with ANT, the other component of mPTP (Figure 6). These data indicated that sevoflurane anesthesia was able to induce a CypD-dependent mitochondrial dysfunction and impairment of neurogenesis, leading to cognitive impairment in young mice. Previous studies have shown that CypD may be associated with AD neuropathogenesis (Wang et al., 2009; Zhu et al., 2013; Adiele and Adiele, 2016) and CypD deficiency may have a protective effect on mitochondrial dysfunction and cognitive impairment in old AD mice (Du et al., 2008, 2011, 2014). However, our studies demonstrated, for the first time, that CypD also contributed to the developmental anesthesia neurotoxicity. Moreover, CypD could be a potential target for the prevention and treatment of anesthesia neurotoxicity in the developing brain. Specifically, the identification of the role of CypD in the developmental anesthesia neurotoxicity could serve the following purposes: (1) clinically, we may assess whether we can use CypD as a biomarker of the developmental anesthesia neurotoxicity; (2) translationally, we should determine whether we can use RNAi or CRISP/cas9 to modify the CypD gene in order to prevent or treat developmental anesthesia neurotoxicity; (3) mechanistically, we could investigate whether the separation of CypD with ANT can lead to neurotoxicity in developing brain.

In current studies, we elucidated the potential role of CypD in anesthesia neurotoxicity. Specifically, the sevoflurane anesthesia was able to increase the levels of CypD. However, the exact mechanisms by which the sevoflurane anesthesia increased the CypD levels remain unknown at the present. We have hypothesized that the elevation of CypD could be due to the imbalance of CypD and ANT or VDAC, the other components of mPTP, and the overmatched CypD can dissociate from the binding with ANT or VDAC, leading to the elevation of CypD. Future studies to test this hypothesis are warranted by asking whether the scavenger of CypD is able to mitigate the anesthesia-induced elevation of CypD *in vitro* and *in vivo*.

Note that we used 60% oxygen concentration in mice in both the control condition group and the sevoflurane anesthesia group, which could serve as a confounding factor in the studies of anesthesia on mitochondrial dysfunction since hyperoxia itself was able to cause mitochondrial stress. We, therefore, performed *in vitro* NPCs studies, using a normal oxygen concentration (21%) in culture condition. We illustrated that sevoflurane anesthesia was able to cause a time-dependent increase in levels of CypD, mitochondrial dysfunction (e.g., opening of mPTP), and impairment of neurogenesis in the NPCs harvested from the WT mice, but not in the NPCs harvested from the CypD KO mice. Finally, the *in vitro* hypothesis generation studies showed that sevoflurane could potentially increase the levels of mitochondrial CypD by releasing its binding with ANT.

Knockout of cyclophilin D has been shown to stabilize mitochondrial function and mitigate the cognitive impairment in old AD transgenic mice (Du et al., 2008, 2011, 2014). Moreover, KO of CypD can inhibit mPTP opening and attenuate arrhythmogenesis in the heart of rats (Gordan et al., 2016). Consistently, we showed that KO of CypD could attenuate the sevoflurane anesthesia-induced mitochondrial dysfunction, impairment of neurogenesis, and cognitive impairment *in vitro* and *in vivo*. However, the current findings were different from the results obtained from the previous studies in that (1) the present studies focused on developmental anesthesia neurotoxicity; and (2) the studies illustrated that KO of CypD attenuated the relatively acute changes including the anesthesia-induced mitochondrial dysfunction, impairment of neurogenesis, and cognitive impairment *in vivo* in young mice and *in vitro* in NPCs.

The studies have several limitations. First, we only used young mice but not the allocated equal number of female and male mice in each group in the present studies because it is difficult to identify the sex of mice at the age of P6. However, the objective of the studies was to determine the role of CypD in the developmental anesthesia neurotoxicity but not the sex-dependent changes. We will use the established system to determine the potential sex-dependent effects in the future. Second, the WT mice were C57 strain, and the CypD KO mice were B6 strain in the present study. The difference in the cognitive function between the WT mice and CypD KO mice following the sevoflurane anesthesia could be due to the strain difference between the WT and CypD KO mice (Brooks et al., 2005). However, the CypD KO mice with C57 strain are not available at present, and the previous and current studies demonstrated that there was no difference in the baseline level of cognitive function between WT C57 strain mice and CypD KO B6 strain mice [(Du et al., 2008; Zhang et al., 2017) and Figure 4 of the current study]. Moreover, our studies also demonstrated the difference in mitochondrial function and neurogenesis between the WT and CypD KO mice in addition to the cognitive function. Finally, we did not see a significant difference in the hippocampus ROS and ATP baseline levels between the WT C57 strain and CypD KO B6 strain mice in current studies (Supplementary Figures 1A,B). All of these findings suggest that it was likely CypD, but not the B6 strain of the mice, that contributed to the sevoflurane-induced mitochondrial dysfunction, impairment of neurogenesis, and cognitive impairment *in vitro* and *in vivo*. Nevertheless, future studies using the same strain of WT and CypD KO mice to further test the hypothesis generated in the current studies are warranted.

## Conclusion

**FIGURE 7:**
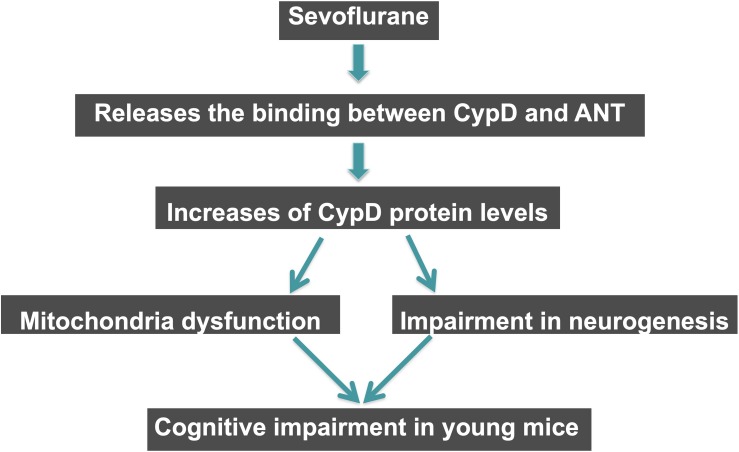
Hypothesized pathway of CypD associated sevoflurane-induced cognitive impairment in young mice. Sevoflurane increases levels of CypD via reducing the binding of CypD with ANT. The increased CypD then causes mitochondrial dysfunctions and impairment of neurogenesis, eventually leading to cognitive impairment in young mice.

In conclusion, in this proof of concept and hypothesis generation research, we found that the sevoflurane anesthesia induced a CypD-dependent mitochondrial dysfunction, impairment of neurogenesis, and cognitive impairment *in vivo* in young mice and *in vitro* in NPCs. Sevoflurane might cause anesthesia neurotoxicity and cognitive impairment via enhancing the levels of CypD in the hippocampus tissues of young mice, and sevoflurane could increase the CypD levels by releasing the binding of CypD with ANT (Figure 6). These findings suggest that CypD may contribute to the developmental anesthesia neurotoxicity and would facilitate the mechanistic investigation of neurotoxicity in the developing brain (Figure 7). Moreover, regulating CypD levels could be one of the targeted interventions of anesthesia neurotoxicity and neurobehavioral deficits in young mice and children, pending further investigation.

## Data Availability Statement

All datasets generated for this study are included in the article/Supplementary Material.

## Ethics Statement

The animal study was reviewed and approved by the Massachusetts General Hospital Standing Committee on Animals (Boston, MA, United States).

## Author Contributions

YZ, PL, FL, and NL conducted the experiments. YZ, JZ, and ZX designed the research. YZ, FL, and NL analyzed the data. YZ and ZX wrote the manuscript. All authors reviewed, edited, and approved the manuscript.

## Conflict of Interest

The authors declare that the research was conducted in the absence of any commercial or financial relationships that could be construed as a potential conflict of interest.

## Abbreviations

ANOVA: analysis of variance
ANT: adenine nucleotide translocase
ATP: adenosine-5′-triphosphate
BrdU: 5′-bromo-2′-deoxyuridine
calcein AM: calcein acetoxymethyl ester
CsA: cyclosporine A
CypD: cyclophilin D
DG: dentate gyrus
EdU: 5-ethynyl-2′-deoxyuridine
JC-1: 5,5′,6,6′-tetrachloro-1,1′,3,3′ tetraethylbenzimidazolylcarbocyanine iodide
MMP: mitochondrial membrane potential
mPTP: mitochondrial permeability transition pore
MWM: Morris Water Maze
NPCs: neural progenitor cells
Ppif: peptidyl-prolyl cis-trans isomerase F
ROS: reactive oxygen species
rpm: revolutions per minute
SD: standard deviation
SVZ: subventricular zone
VDAC: voltage-dependent anion channel
WT: wild type.
