# Supplementary material for: Cyclophilin D Contributes to Anesthesia Neurotoxicity in the Developing Brain
Source: Front Cell Dev Biol. 2020 Feb 11;7:396. doi: 10.3389/fcell.2019.00396 (PMC7026027; doi:10.3389/fcell.2019.00396)
Supplement: Supplementary file 1 [file Data_Sheet_1.docx]

Supplemental Materials

Cyclophilin D contributes to anesthesia neurotoxicity in the developing brain

Yiying Zhang, Pan Lu, Feng Liang, Ning Liufu, Yuanlin Dong,

Jialin Charles Zheng and Zhongcong Xie

**MATERIALS AND METHODS**

**Mice anesthesia**

We anesthetized the young mice with 3% sevoflurane for 2 hours daily at postnatal day 6 (P6), P7, and P8 based on the literature that the brain growth spurt reaches the peak in humans around birth, and P7 in rodents [([Dobbing and Sands, 1979](#_ENREF_1)), reviewed in ([Semple et al., 2013](#_ENREF_3))], and based on the data of our previous studies showing that anesthesia with 3% sevoflurane 2 hours daily on P6, P7, and P8 caused neuroinflammation, Tau phosphorylation and cognitive impairment in the mice ([Shen et al., 2013](#_ENREF_4); [Tao et al., 2014](#_ENREF_5)). Given it is difficult to identify the sex of the mice at P6 and the objective of the present studies was not to determine the sex-dependent effects, we did not allocate the equal number of female or male mice in each of the experimental or control group. Instead, the mixture of female and male young mice was used in the studies (P6 mice from both sexes). The mice were obtained from our breeding. The mice were housed in a temperate- and humidity-controlled environment (20 - 22°C; 12-hour light: dark on a reversed light cycle) with free access to water and food. We randomly assigned the mice to either the sevoflurane anesthesia group or the control condition group. The mice in the sevoflurane anesthesia group received 3% sevoflurane plus 60% oxygen (balanced with nitrogen) at P6, P7, and P8 as performed in our previous studies ([Lu et al., 2017](#_ENREF_2); [Shen et al., 2013](#_ENREF_4); [Tao et al., 2014](#_ENREF_5); [Zhang et al., 2015](#_ENREF_6)). The mice in the control condition group received 60% oxygen (balanced with nitrogen) at an identical flow rate in similar chambers according to our previous studies ([Lu et al., 2017](#_ENREF_2); [Shen et al., 2013](#_ENREF_4); [Tao et al., 2014](#_ENREF_5); [Zhang et al., 2015](#_ENREF_6)). We monitored the anesthetic and oxygen concentrations continuously with a gas analyzer (Ohmeda; GE Healthcare, Tewksbury, MA). We controlled the temperature of the anesthetizing chamber by the DC Temperature Control System (FHC, Bowdoinham, ME), which is a feedback-based system for monitoring and controlling temperature, to maintain the rectal temperature of the mice as 37° ± 0.5°C. Our previous studies ([Lu et al., 2017](#_ENREF_2); [Shen et al., 2013](#_ENREF_4); [Tao et al., 2014](#_ENREF_5); [Zhang et al., 2015](#_ENREF_6)) showed that 3% sevoflurane for two hours did not significantly change the values of pH, the partial pressure of oxygen, or partial pressure of carbon dioxide as compared with the control group. The mortality rate of mice was less than 1% in the studies*.* The experiments were performed blindly, e.g., the persons who performed the behavior, and cellular studies did not know the condition of anesthesia or control in the mice or cells.

**5'-bromo-2'-deoxyuridine (BrdU) injection for the assessment of neurogenesis**

BrdU solution (5 mg/ml) was made using sterile saline for pH of 7.4 and stored at 20 °C*.* One group of mice was euthanized on P8 immediately after the sevoflurane anesthesia for the assessment of neurogenesis. The rest of the mice were injected BrdU every four days and euthanized on P36 to assess the potential later effects of sevoflurane on the neurogenesis. Note, the mice that received BrdU injections were only used for hippocampus immunofluorescence staining, but not for the behavioral or other studies.

**Harvest of brain tissues**

We used half of the harvested hippocampus tissues for the immunohistochemistry staining and half of them for the Western blots, reactive oxygen species (ROS), and Adenosine triphosphate (ATP) measurement. We used different mice and harvested their hippocampus tissues to obtain the mitochondria for the studies to detect mitochondrial membrane potential (MMP).

**Mitochondria isolation**

Given the volume of the harvested mitochondria from the hippocampus tissues of each of P8 mice was small, we mixed the hippocampus tissues of 6 of P8 mice to isolate the mitochondria, and then divided these tissues into three samples. The hippocampus tissues obtained on P8 were added to 800µL of PBS, then the tissues were disrupted by using a Dounce homogenizer on ice. The homogeneous suspension was centrifuged at 1,000 × g for 3 min at 4°C, and the supernatant was discarded. Next, we suspended the pellet into 800 µL of BSA/reagent solution in a tube, vortexed the tube at medium speed for 5 seconds, and incubated the tube on ice for 2 min. Then, we added 10µL of mitochondria isolation reagent B into the tube and vortexed the tube at maximum speed for 5 seconds, following by incubating the tube on ice for 5 min, vortexing at maximum speed every minute. We added 800µL of mitochondria isolation reagent C into the tube and centrifuged the tube at 700 × g for 10 min at 4°C, and transferred the supernatant into a new tube and centrifuged the tube at 3,000 × g for 15 min at 4°C. Finally, we removed the supernatant into another new tube and maintained the tube containing the mitochondrial pellet on ice for the Mass spectrometry studies and the assessment of mitochondrial membrane potential.

**Western blots analysis**

The harvested hippocampus tissues were homogenized on ice using an immunoprecipitation buffer (10 mM Tris-HCl, pH 7.4, 150 mM NaCl, 2 mM EDTA, 0.5% Nonidet P-40) plus protease inhibitors (1 μg/ml aprotinin, 1 μg/ml leupeptin, 1 μg/ml pepstatin A). The lysates were collected, centrifuged at 13,000 rpm for 15 min, and quantified for the amount of total proteins by a bicinchoninic acid protein assay kit (Pierce, Iselin, NJ). The harvested hippocampus tissues or NPCs were subjected to Western blot analyses as described in our previous studies ([Zhang et al., 2013](#_ENREF_8); [Zhang et al., 2014](#_ENREF_9)). Each band in the Western blot represented an independent experiment. The quantification of Western blot, averaged from six independent experiments, was accomplished as described in other studies ([Zhang et al., 2010](#_ENREF_7); [Zhang et al., 2013](#_ENREF_8); [Zhang et al., 2012](#_ENREF_10); [Zhang et al., 2011](#_ENREF_11)). Briefly, the intensity of signals was analyzed using the Image-J (NIH Image 1.62, Bethesda, MD). The Western blots were quantified using two steps. First, β-actin levels were used to normalize (e.g., determining the ratio of CypD levels to β-actin levels) for any loading differences in total protein amounts. Second, changes in protein levels in different groups were presented as percentages of the corresponding levels in the control conditions.

**Reactive oxygen species (ROS) measurement**

Briefly, NPCs cells were placed in a clear 96-well cell culture plate overnight in the incubator. We then added the 2',7'-dichlorfluorescein-diacetate (DCFH-DA)/media solution to the cells. The DCFH-DA loaded NPCs cells were then exposed to 4.1% sevoflurane for 6h. The treated cells were lysed by adding 100 μL of cell lysis buffer and were mixed thoroughly and incubated for five min at room temperature. 150 μL of the mixture was transferred to each well of a 96-well plate to be used for fluorescence measurement. For the *in vivo* studies, the harvested brain tissues were homogenized on ice using 1% triton-100 in PBS buffer plus protease inhibitors (1 μg/ml aprotinin, 1 μg/ml leupeptin, 1 μg/ml pepstatin A). The lysates were collected, centrifuged at 10,000 rpm for 5 min, and quantified for total proteins by a bicinchoninic acid protein assay kit (Pierce, Iselin, NJ). We added 50 μL of each sample (triplicate) to a black 96-well plate and 50 μL of catalyst solution provided, and then incubated the mixture for 5 min at room temperature. We added 100 μL of dichlorodihydrofluorescin (DCFH) solution included in the kit to the samples, and incubated the mixture at room temperature for 20 min away from light. Finally, the fluorescence was read with a fluorometric plate reader at 480 nm/530 nm.

**Measurement of mitochondrial membrane potential (MMP)**

Specifically, the treated NPCs cells and isolated mitochondria from the harvested mice hippocampus tissues were incubated with JC-1 reagents at 37°C for 15 min and washed twice with HBSS. Finally, fluorescence was read with a fluorometric plate reader at red fluorescence (excitation 590 nm, emission 610 nm) and green fluorescence (excitation 490 nm, emission 520 nm) using a fluorescence plate reader. The level of MMP was calculated by the ratio of red:green fluorescence.

**ATP measurement**

Briefly, the harvested mice hippocampus tissue or cell lysis were used to test the ATP levels. The amount of fluorescence was measured, and the levels of ATP in the experimental samples were calculated from the standard curve with a known amount of ATP.

**Sectioning of fresh unfixed tissue using a compresstome**

Briefly, the mouse brain was sectioned with the Compresstome® VF-310-0Z (Precisionary Instruments, Natick, MA) using the protocol provided by the company with modifications. Specifically, the brain tissues were glued onto the cutting plunger of a specimen syringe using Loctite® 404® Quick Set Instant Adhesive. The brain tissues were pulled down into the plunger and embedded with 2% agarose (dissolved in PBS-H-buffer) at 40°C, and the plunger with agarose and brain tissues were placed into a chilling block until the agarose solidified (< 2 min). The buffer tank was filled with cold PBS buffer to maintain the temperature at 0°C. The machine automatically cut the brain tissues into free-floating tissue sections with a thickness of 15 μm. The brain section slices were collected and used for the immunohistochemistry staining.

**Immunohistochemistry**

For BrdU staining, the brain sections from each treatment group at P8 and P36 were collected. We fixed the brain sections using formalin for 30 min at room temperature. We examined the staining sections using a 10 X and 40 X objective and imaged for BrdU and Nestin [the markers of Neural progenitor cells (NPCs)] positive cells. The following antibodies and dilution were used: anti-BrdU (1:200, ab6326, Abcam); anti-Nestin (1:100, [MAB353](https://www.google.com/url?sa=t&rct=j&q=&esrc=s&source=web&cd=1&cad=rja&uact=8&ved=0ahUKEwj4rKKiuJTYAhVNtJQKHUWADdsQFgg8MAA&url=http%3A%2F%2Fwww.emdmillipore.com%2FUS%2Fen%2Fproduct%2FAnti-Nestin-Antibody-clone-rat-401%2CMM_NF-MAB353&usg=AOvVaw0jYzNvd7mODc64zPoeqE5m)**,** Chemicon, Waltham, MA). The regions of interest in the dentate gyrus [outer and inner halves of the granular layer (GCL)] were analyzed using a KEYENCE BZ-9000E all-in-one fluorescence microscope (KEYENCE Corporation of America) and the software provided by the company. The software could analyze different colors corresponding to specific Nestin staining or BrdU staining. Then, the numbers of BrdU-positive cells and BrdU/Nestin-double positive cells in each region per unit area were counted with Image-J (NIH Image 1.62, Bethesda, MD). The mean number of BrdU-positive cells and BrdU/Nestin-double positive cells were calculated from six brain sections for each animal.

**Neural progenitor cells (NPCs) culture**

Specifically, we used hippocampus tissues harvested from 18 or 19-day gestation stage of WT or CypD KO mice. Cesarean sections were performed to pull out the mice embryos, and the mice were decapitated in a 100 mm dish of phosphate-buffered saline (PBS). The hippocampus tissues were dissected out and put into a 15 ml tube with 2.5 ml “complete” proliferation media, triturated 10 - 15 times using a 1,000 μl plastic pipette tip and filtered through a 70 μm cell strainer to obtain suspended single NPC. One million cells were plated in each well of a six-well plate containing 2.5 ml of "complete" proliferation media. NPCs were passaged twice (every seven days) before use. For passaging the cells, media and cells were removed and placed into a 50 ml centrifuge tube to remove adherent NPCs and dead cells. Only non-adherent proliferating NPCs were collected. The NPCs were centrifuged at 3,000 rapid per minute (rpm) for 5 min. The medium was removed, and the cells were re-suspended in 3 ml of "complete" proliferation media. The cells were then triturated 10 - 15 times using a 1,000 μl plastic pipette tip and then plated at one million cells in 2.5 ml of “complete” proliferation media in each well of a six-well plate.

**NPCs and H4 cells culture and treatment.**

One million cells were plated in each well of a six-well plate containing 2.5 ml of “complete” proliferation media, generated with a final concentration of 20 ng/ml of hEGF, 100 units/ml penicillin, and 100 μg/ml streptomycin. The NPCs were treated with 4.1% sevoflurane plus 21% O_2_ and 5% CO_2_ as described in previous studies with modifications ([Zhang et al., 2013](#_ENREF_8); [Zhang et al., 2014](#_ENREF_9)). The NPCs were treated from 3 to 6 hours to assess the time-dependent changes of CypD levels. Given the findings that 3 hours of treatment with 4.1% sevoflurane did not cause cell death ([Zimering et al., 2016](#_ENREF_13)), the NPCs were treated for 3 hours for the measurement of [reactive oxygen species](https://www.google.com/url?sa=t&rct=j&q=&esrc=s&source=web&cd=1&cad=rja&uact=8&ved=0ahUKEwiqmcfro5TYAhWIVZQKHTg5CVgQFggpMAA&url=https%3A%2F%2Fen.wikipedia.org%2Fwiki%2FReactive_oxygen_species&usg=AOvVaw3ndxpmP3Ys0jI3--vizf5-) (ROS), mitochondrial permeability transition pore (mPTP), mitochondrial membrane potential (MMP), adenosine-5'-triphosphate (ATP). The NPCs were treated for 6 hours for [5-Ethynyl-2'-deoxyuridine](http://en.wikipedia.org/wiki/5-Ethynyl-2%27-deoxyuridine) (EdU) staining because the same sevoflurane anesthesia was able to decrease proliferation of NPCs ([Zhang et al., 2013](#_ENREF_8)). The H4 naïve human neuroglioma cells were used in the co-immunoprecipitation studies because we were able to have large amounts of the H4 cells for the studies. The H4 naïve cells were treated with 4.1% sevoflurane with 6 hours.

**Labeling NPCs using** [**5-Ethynyl-2'-deoxyuridine**](http://en.wikipedia.org/wiki/5-Ethynyl-2%27-deoxyuridine) **(EdU)** **and quantification.**

The NPCs were cultured in FluoroDish (World Precision Instruments, Inc., Sarasota, FL), coated with Poly-D-lysine (PDL) with laminin, with “complete” proliferation media overnight in the incubator. Next day, NPCs were incubated with EdU and “complete” proliferation media (final concentration 10 μM) for five min before the treatment of 4.1% sevoflurane for 6 hours. At the end of the treatment, the NPCs were fixed with 4% paraformaldehyde for 15 min at room temperature and washed with 3% bovine serum albumin (BSA) in PBS twice, and then incubated within 0.2% TritonX-100 (Sigma) in PBS for 20 min at room temperature. The cells were then washed twice with PBS and incubated with 500 μl Click-iT™ reaction cocktail for 30 min at room temperature. Finally, the NPCs were washed twice with 3% BSA in PBS and used for staining. We randomly selected eight fields per FluoroDish and took the images using a KEYENCE BZ-9000E all-in-one fluorescence microscope (KEYENCE corporation of America) under a 20 X objective microscope lens. In the flow cytometry studies, after the staining and collection of the cells, we used the BD LSRFortessa™ X-20 flow cytometers (BD Biosciences) to analyze the cells.

**Flow Cytometric analysis of mPTP opening.**

The cell culture media were treated with 4% sevoflurane for 2 hours before being added to the cells. The cells were treated with 4% sevoflurane for another one hour. Under normal conditions, the non-fluorescent acetoxymethyl ester (AM) of calcein dye (calcein AM) and cobalt can enter the cell. The acetoxymethyl ester (AM) groups are cleaved from calcein via non-specific esterase, and calcein can then show fluorescence signals in both the cytosol and mitochondria. Cobalt can quench the cytosolic calcein signal. However, cobalt cannot enter healthy mitochondria freely, and therefore cannot quench the mitochondrial calcein signal. When the opening of mPTP occurs, cobalt enters through the pore and subsequently quenches the mitochondrial calcein signal. Flow cytometry was used to detect the amounts of cells that exhibit quenched calcein signals inside the mitochondria. The location of the curves indicates the amount of such cells, which suggests the opening of mPTP. Ionomycin was used as a positive control for the opening of mPTP in the experiments. Dead cells and debris were excluded from analysis by gates set on forward, and side-angle light scatter.

**Reverse transcriptase polymerase chain reaction (RT-PCR).** Ribonucleic acid (RNA) was isolated from WT NPCs. CypD messenger RNA levels were determined and standardized using glyceraldehyde 3-phosphate dehydrogenase (GAPDH) as an internal control. Primers of mouse Ppif (CypD)(ID No., PPM28868B-200) and mouse GAPDH (ID No., QT01658692) were purchased from Qiagen. PCR reactions were performed at 50 °C for 30 min, 95 °C for 15 min. This was followed by 50 cycles of 94 °C for 15 seconds, 55 °C for 30 seconds, 72 °C for 30 s, and finally, 95 °C for 15 seconds and 55 °C for 15 seconds.

[**Immunocytochemistry**](https://www.google.com/url?sa=t&rct=j&q=&esrc=s&source=web&cd=2&cad=rja&uact=8&ved=0ahUKEwiE7PfT5YfYAhWLw4MKHZ-NBhoQFgguMAE&url=https%3A%2F%2Fen.wikipedia.org%2Fwiki%2FImmunocytochemistry&usg=AOvVaw0C8hiegIxD1svw3EXG9fTb) **staining.** The antibody used in the experiments included anti-Nestin antibody (1:200, Abcam); anti-CypD (1:500, Abcam); anti-ANT(1:500, Abcam), and DAPI (4',6-Diamidino-2-Phenylindole, Dihydrochloride). For the EdU studies, the NPCs were incubated with 10 μg/ml Hoechst 33342 (Trihydrochloride, Trihydrate) after the secondary antibody, and then analyzed in mounting medium under a 20 X objective lens fluorescence microscope.

**Co-immunoprecipitation of CypD and ANT.**

We used H4 cells, but not NPCs, in the studies because we were able to harvest larger amount of H4 cells for the co-immunoprecipitation studies to assess the binding of CypD and ANT. Briefly, H4 naïve cells extracts at 200 μg of protein per sample were precleared with protein G beads and then mixed with 2 μg of an ANT antibody or control non-immune mouse Ig immobilized on protein G beads in an “immunoprecipitation buffer” (20 mM Tris-HCl, pH 7.5, 150 mM NaCl, 1.5% Nonidet P-40) supplemented with protease inhibitors. The reactions were incubated overnight at 4 °C. Immunocomplexes were washed in an “immunoprecipitation wash buffer” (100 mM Tris-HCl, pH 7.5, 100 mM NaCl, 0.1% Triton X-100) and resuspended in Laemmli buffer. Elution of immunocomplexes with Laemmli sample buffer and heat-denaturation were performed immediately prior to one-dimensional gel electrophoresis. Proteins were separated on 4-12% SDS–polyacrylamide gels and electroblotted onto PVDF membranes. The Western blots were then probed with antibodies against ANT or CypD. Western blot membranes were blocked with 5% nonfat milk and incubated with antibodies anti-ANT (Abcam) or anti-CypD (Abcam) overnight at 4 °C.

**Morris Water Maze (MWM) studies**

Briefly, a round steel pool, 150 cm in diameter and 60 cm in height, was filled with water to a height of 1.0 cm above the top of a 10-cm diameter platform. The pool was covered with a black curtain and was located in an isolated room with three visual cues on the wall of the pool. Water was kept at 20 °C and opacified with titanium dioxide. At P28, the mice were tested in the MWM for three trials per day for seven days. Each mouse was placed in the pool to search for the platform. The starting points were random for each mouse. After the mouse found the platform, it stayed on the platform for 15 seconds. The time for each mouse to find the platform was defined as escape latency. If the mouse did not find the platform within 90 seconds, the mouse was gently guided to the platform and allowed to stay on it for 15 seconds. A video tracking system recorded the swimming motions of the animals, and the data were analyzed using motion-detection software for the MWM. At the end of the reference training (P34), the platform was removed from the pool, and the mouse was placed in the random quadrant. Each mouse was allowed to swim for 90 seconds, and the number of times the mouse swam across the platform area was recorded (platform crossing times). Both escape latency and platform crossing times were recorded and served as the measurement of cognition. Mouse body temperature was maintained by active heating as described before ([Shen et al., 2013](#_ENREF_4); [Tao et al., 2014](#_ENREF_5); [Zheng et al., 2013](#_ENREF_12)). Specifically, after every trial, each mouse got dry under a heat lamp before returning to its cage. The mice in the MWM studies were only used for cognition studies.

**Supplemental Figures**.


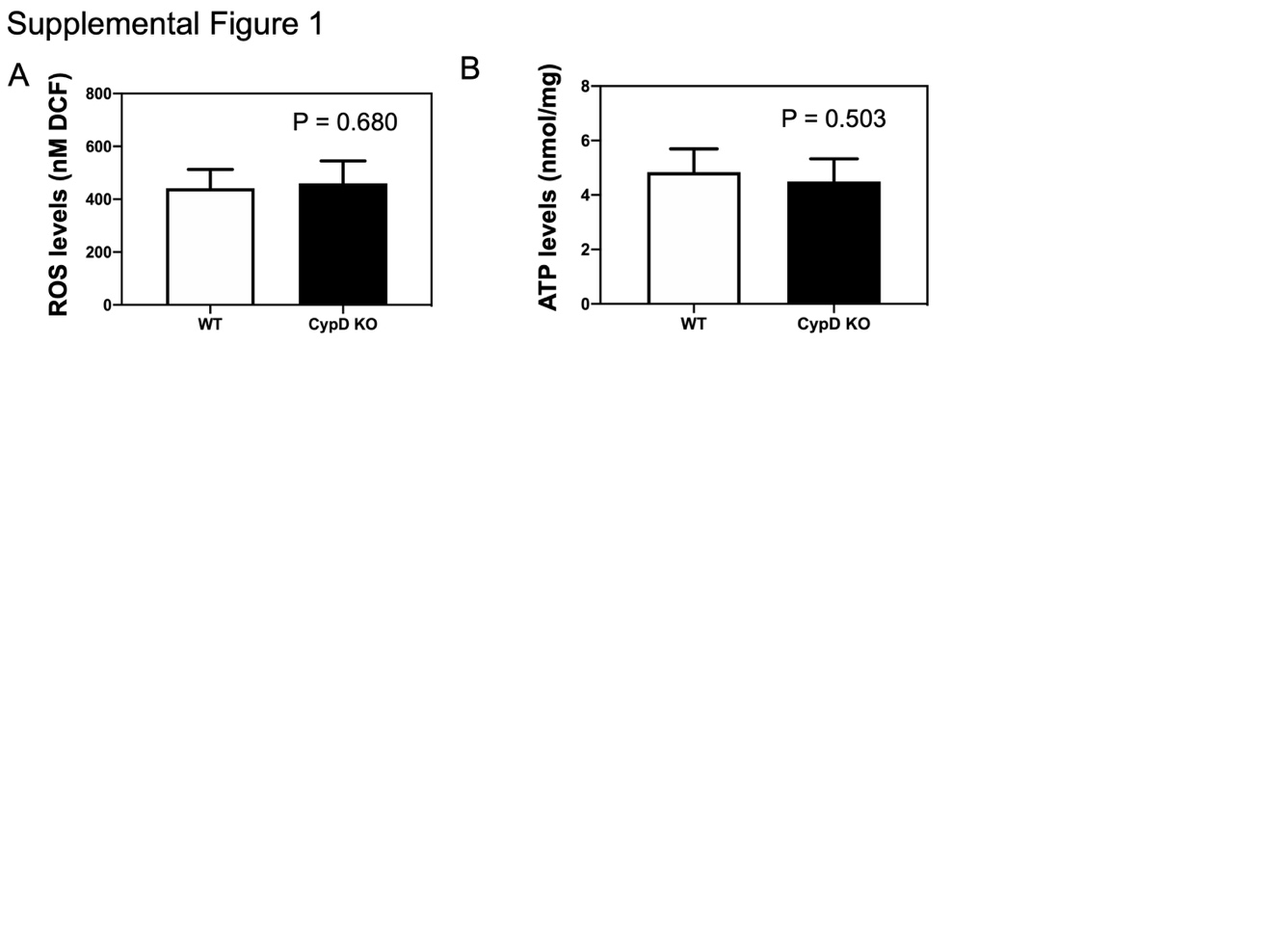


Supplemental Figure 1. **A**. ROS baseline levels in hippocampus tissues of WT mice (white bar) and CypD KO mice (black bar). There was no significant difference in the ROS levels between the WT mice and the CypD KO mice (P = 0.680, Student’s *t* test, N = 6). **B**. ATP baseline levels in hippocampus tissues of WT mice (white bar) and CypD KO mice (black bar). There was no significant difference in the ATP levels between the WT mice and the CypD KO mice (P = 0.503, Student’s *t* test, N = 6).


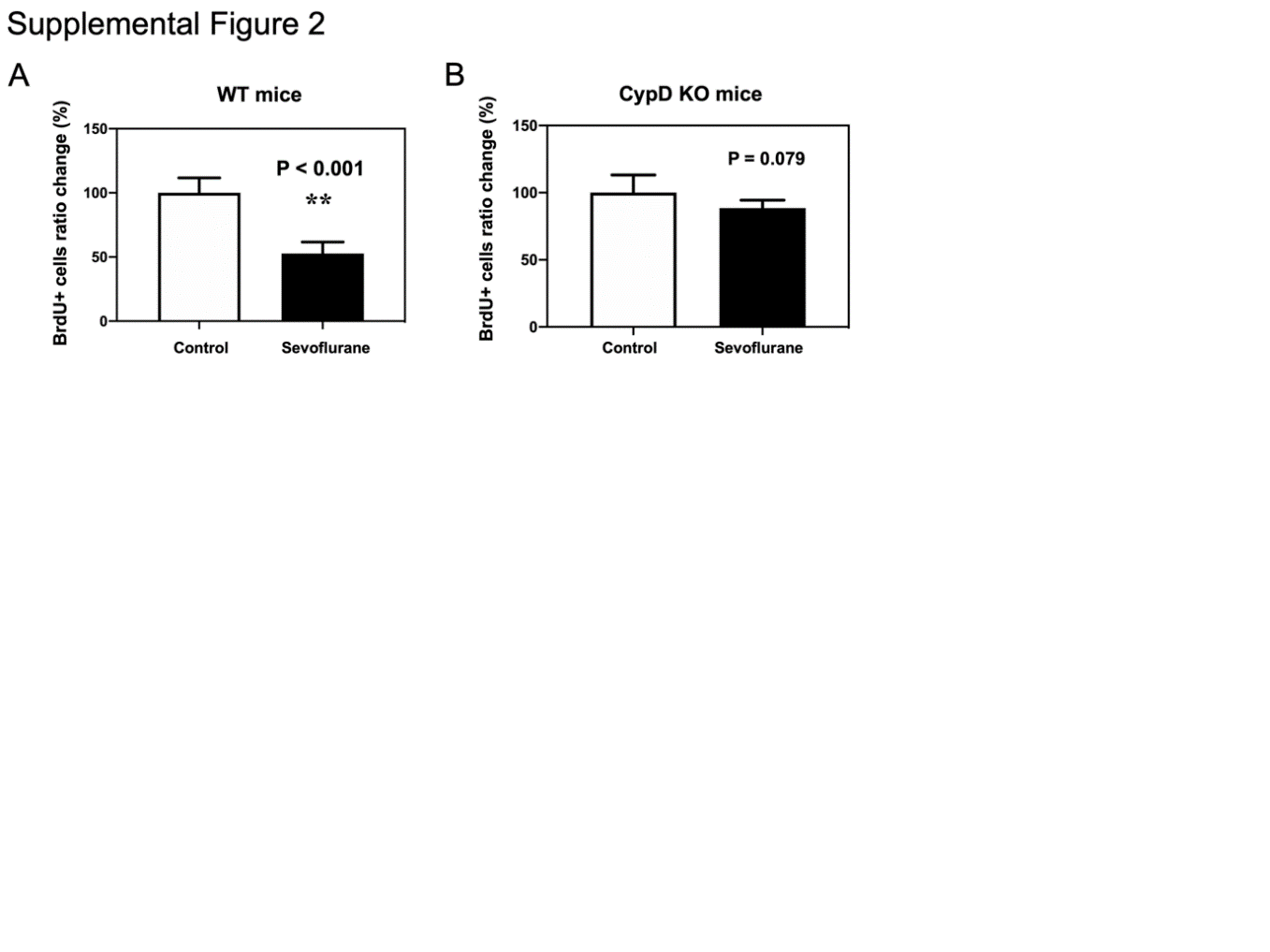


Supplemental Figure 2. **A**. Quantification of BrdU positive cells (ratio change) in the control condition (white bar) and sevoflurane anesthesia (black bar) in the WT mice (**P < 0.001, Student’s *t* test, N = 6). The sevoflurane anesthesia decreased the ratio of change in the BrdU+ cells as compared to the control condition. **B**. Quantification of BrdU positive cells (ratio change) in control condition (white bar) and sevoflurane anesthesia (black bar) in the CypD KO mice (**P = 0.079, Student’s *t* test, N = 6). The sevoflurane anesthesia did not significantly alter the ratio of change in the BrdU+ cells as compared to the control condition.

References

Dobbing, J., and Sands, J. (1979). Comparative aspects of the brain growth spurt. Early Hum Dev 3, 79-83.

Lu, H., Liufu, N., Dong, Y., Xu, G., Zhang, Y., Shu, L., Soriano, S.G., Zheng, H., Yu, B., and Xie, Z. (2017). Sevoflurane Acts on Ubiquitination-Proteasome Pathway to Reduce Postsynaptic Density 95 Protein Levels in Young Mice. Anesthesiology 127, 961-975.

Semple, B.D., Blomgren, K., Gimlin, K., Ferriero, D.M., and Noble-Haeusslein, L.J. (2013). Brain development in rodents and humans: Identifying benchmarks of maturation and vulnerability to injury across species. Prog Neurobiol 106-107, 1-16.

Shen, X., Dong, Y., Xu, Z., Wang, H., Miao, C., Soriano, S.G., Sun, D., Baxter, M.G., Zhang, Y., and Xie, Z. (2013). Selective anesthesia-induced neuroinflammation in developing mouse brain and cognitive impairment. Anesthesiology 118, 502-515.

Tao, G., Zhang, J., Zhang, L., Dong, Y., Yu, B., Crosby, G., Culley, D.J., Zhang, Y., and Xie, Z. (2014). Sevoflurane induces tau phosphorylation and glycogen synthase kinase 3beta activation in young mice. Anesthesiology 121, 510-527.

Zhang, J., Dong, Y., Zhou, C., Zhang, Y., and Xie, Z. (2015). Anesthetic sevoflurane reduces levels of hippocalcin and postsynaptic density protein 95. Molecular neurobiology 51, 853-863.

Zhang, Y., Dong, Y., Wu, X., Lu, Y., Xu, Z., Knapp, A., Yue, Y., Xu, T., and Xie, Z. (2010). The mitochondrial pathway of anesthetic isoflurane-induced apoptosis. J Biol Chem 285, 4025-4037.

Zhang, Y., Dong, Y., Zheng, H., Shie, V., Wang, H., Busscher, J.J., Yue, Y., Xu, Z., and Xie, Z. (2013). Sevoflurane inhibits neurogenesis and the Wnt-catenin signaling pathway in mouse neural progenitor cells. Current molecular medicine 13, 1446-1454.

Zhang, Y., Pan, C., Wu, X., Dong, Y., Culley, D.J., Crosby, G., Li, T., and Xie, Z. (2014). Different effects of anesthetic isoflurane on caspase-3 activation and cytosol cytochrome c levels between mice neural progenitor cells and neurons. Frontiers in cellular neuroscience 8, 14.

Zhang, Y., Xu, Z., Wang, H., Dong, Y., Shi, H.N., Culley, D.J., Crosby, G., Marcantonio, E.R., Tanzi, R.E., and Xie, Z. (2012). Anesthetics isoflurane and desflurane differently affect mitochondrial function, learning, and memory. Ann Neurol 71, 687-698.

Zhang, Y., Zhen, Y., Dong, Y., Xu, Z., Yue, Y., Golde, T.E., Tanzi, R.E., Moir, R.D., and Xie, Z. (2011). Anesthetic propofol attenuates the isoflurane-induced caspase-3 activation and Abeta oligomerization. PloS one 6, e27019.

Zheng, H., Dong, Y., Xu, Z., Crosby, G., Culley, D.J., Zhang, Y., and Xie, Z. (2013). Sevoflurane anesthesia in pregnant mice induces neurotoxicity in fetal and offspring mice. Anesthesiology 118, 516-526.

Zimering, J.H., Dong, Y., Fang, F., Huang, L., Zhang, Y., and Xie, Z. (2016). Anesthetic Sevoflurane Causes Rho-Dependent Filopodial Shortening in Mouse Neurons. PloS one 11, e0159637.
